# The relevance of lead prioritization: a B2B lead scoring model based on machine learning

**DOI:** 10.3389/frai.2025.1554325

**Published:** 2025-03-07

**Authors:** Laura González-Flores, Jessica Rubiano-Moreno, Guillermo Sosa-Gómez

**Affiliations:** ^1^Universidad Panamericana, Facultad de Ciencias Económicas y Empresariales, Zapopan, Jalisco, Mexico; ^2^Facultad de Ciencias Administrativas y Comerciales, Universidad de Ciencias Aplicadas y Ambientales, Bogotá, Colombia

**Keywords:** lead scoring, digital marketing, B2B sales, business-to-business, marketing automation, lead qualification, lead conversion, CRM

## Abstract

In business-to-business (B2B) companies, marketing and sales teams face significant challenges in identifying, qualifying, and prioritizing a large number of leads. Lead prioritization is a critical task for B2B organizations because it allows them to allocate resources more effectively, focus their sales force on the most viable and valuable opportunities, optimize their time spent qualifying leads, and maximize their B2B digital marketing strategies. This article addresses the topic by presenting a case study of a B2B software company's development of a lead scoring model based on data analytics and machine learning under the consumer theory approach. The model was developed using real lead data generated between January 2020 and April 2024, extracted from the company's CRM, which were analyzed and evaluated by fifteen classification algorithms, where the results in terms of accuracy and ROC AUC showed a superior performance of the Gradient Boosting Classifier over the other classifiers. At the same time, the feature importance analysis allowed the identification of features such as “source” and “lead status,” which increased the accuracy of the conversion prediction. The developed model significantly improved the company's ability to identify high quality leads compared to the traditional methods used. This research confirms and complements existing theories related to understanding the application of consumer behavior theory and the application of machine learning in the development of B2B lead scoring models. This study also contributes to bridging the gap between marketers and data scientists in jointly understanding lead scoring as a critical activity because of its impact on overall marketing strategy performance and sales revenue performance in B2B organizations.

## 1 Introduction

In business-to-business (B2B) sales processes, marketing and sales teams face the challenge of identifying, qualifying, and prioritizing leads. Lead qualification is a critical task because it impacts conversion rates and maximizes the effectiveness of marketing and sales efforts and strategies (Priya, 2020). Digital marketing strongly emphasizes the need to address potential customers in a personalized manner in the B2B segment (Espadinha-Cruz et al., 2021), this challenges the management of potential customers in organizations, motivating them to develop a greater understanding of potential customers by viewing them as unique individuals, with particular interests, needs, aspirations and behaviors (Bondarenko et al., 2019), however in the B2B segment the process of managing potential customers is often ineffective and the conversion of potential customers into real customers is an issue that is not entirely clear (Espadinha-Cruz et al., 2021).

The industrial revolution 4.0 has been transforming industry and economics by generating major trends such as big data, the application of artificial intelligence and the digitization of markets (Gouveia and Costa, 2022; Khan and Iqbal, 2020). This digitization of markets is redefining the way customer relationships are managed and built, the way companies communicate their value offering, and the way customers buy in B2B markets (Hofacker et al., 2020). In this digital, dynamic and constantly evolving environment there is a marked trend toward an economic system in which customer prioritization may dominate business relationships (Libai et al., 2020). Organizations in the B2B segment that usually operate with seven-step consultative sales models (Moncrief and Marshall, 2005), that require more processes to complete transactions, that their sales take longer to close than in a B2C market (D'Haen and Van den Poel, 2013) and where their sales representatives have highly committed times with multiple activities, face the difficult challenge of adapting and differentiating themselves (Zaif and Cerchia, 2019).

Sales representatives often feel overwhelmed (D'Haen et al., 2013) when faced with qualifying and following up on a large number of leads from different sources and generated from the various marketing strategies and campaigns, because they usually have their time committed to different activities such as customer acquisition, customer retention, and non-sales activities, making it challenging to allocate time to lead qualification and follow-up activities (Sabnis et al., 2013).

The task of lead qualification often becomes difficult to achieve because sales reps do not have the initial information to determine how viable one sales opportunity is in relation to the others (Priya, 2020), so that they can establish the order of attention they should give to leads according to their likelihood of conversion, situation that frequently derives in late leads contacts (Nygård and Mezei, 2020), arbitrary decisions (D'Haen et al., 2013), based on intuition when selecting leads to work with (Järvinen and Taiminen, 2016), causing waste of resources, inaccurate sales forecasts and lost sales (Monat, 2011).

The purpose of this paper is to find a way to qualify and prioritize leads in a standardized and automatic way, as well as to explore how artificial intelligence influences B2B lead classification tasks. To this end, the authors propose a lead scoring model based on supervised machine learning techniques to predict and prioritize leads in a B2B company that provides software solutions for product design and manufacturing. This company needed a lead scoring model that was not static and allowed marketing and sales teams to determine the order of attention they should give to leads based on their probability of conversion, since there was evidence that sales representatives, as in other B2B organizations, did not have information at an early stage to determine the probability of a lead and often focused on qualifying leads without an established order.

Given the above, this study seeks to explore the relationship between scoring models and lead prioritization, hypothesizing that the implementation of the machine learning based lead scoring model facilitates lead prioritization in B2B markets. The questions that guide this research are: how to identify, in a standardized and automated way, which leads are priority leads in order to optimize the time of sales reps destined to qualify B2B leads; what are the characteristics of the most relevant leads for the lead scoring model; how can artificial intelligence influence the scoring of B2B leads; which supervised machine learning techniques could help predict the probability of lead conversion into business-to-business sales.

This study addresses a case study of a B2B software company for product design and manufacturing that needed a non-static lead scoring model that would allow marketing and sales teams to determine the order of attention to give to prospects based on their likelihood of conversion, as evidence was found that sales representatives, as in other B2B organizations (Nygård and Mezei, 2020; Eitle and Buxmann, 2019; D'Haen and Van den Poel, 2013), did not have information at an early stage to determine the likelihood of a potential customer converting and often focused on scoring leads without an established order. Therefore, the purpose of this article was to develop a lead scoring model based on machine learning techniques to improve the accuracy of identifying high quality customers with the goal of prioritizing leads for sales representatives in a B2B company case study.

An effective lead scoring system is critical because it allows companies to focus on the most valuable leads by increasing the conversion rate (Nygård and Mezei, 2020), makes offer personalization possible, contributes to higher sales team and customer satisfaction (van der Borgh et al., 2020), and ultimately improve companies' sales revenue.

A small amount of academic contributions related to the success of lead scoring systems in B2B sales can be found in the literature (Eitle and Buxmann, 2019; Nygård and Mezei, 2020), only a few researchers have dedicated their research to the development of machine learning models with the aim of facilitating lead scoring and predicting the probability of winning a sale (Espadinha-Cruz et al., 2021).

Predicting the conversion of a B2B lead is a fertile field for research because of the implications this has on sales and revenue for companies. Lead prediction and segmentation are crucial in the B2B segment (Rohaan et al., 2022) and given the modest attention paid to the success of B2B lead scoring models there is a need to expand the amount of research in this area.

The authors begin with the introduction providing the context of the topic, then present the literature review on key concepts such as B2B digital marketing, consumer behavior theory and its influence on lead scoring models, lead scoring models, artificial intelligence and machine learning, and predictive classification models for lead scoring, all of which are relevant topics and closely related to the development of lead scoring models. Then, the methodology is proposed, including the case study of a lead scoring model using machine learning techniques, the results and discussion are presented, then the academic and managerial implications, conclusion and limitations, and future research are discussed.

## 2 Literature review

### 2.1 B2B digital marketing

Digital marketing for the business-to-business or B2B sales segment as it is commonly known can be understood as the adaptive and non-intrusive technology-enabled process by which companies create, communicate and deliver value to their customers. Digital marketing includes a wide variety of strategies and tactics to promote products through digital media and some of its components are content marketing, social media marketing (SMM), mobile marketing, e-commerce, customer data mining (Figueiredo et al., 2021), search engine marketing (SEM), search engine optimization (SEO), affiliate marketing, email marketing, digital display advertising and web analytics, among others (Rosario and Cruz, 2019).

Although little attention has generally been given to the study of the digitization of B2B Marketing (Hofacker et al., 2020), digitization is an increasingly present theme in the way business is done in this segment. The use of artificial intelligence technologies driven by machine learning methods and Big Data have a great impact on advertising and digital marketing (Gao et al., 2023), and on the other hand in terms of lead generation possibly the greatest contribution of artificial intelligence is the ability to target customers in a highly personalized and individualized way (Syam and Sharma, 2018) and the possibility of the creation of lead scoring models.

### 2.2 Marketing automation

The combined use of process design and technology in marketing make marketing automation possible (Lindahl, 2017). Marketing automation is realized by software programs that allow monitoring and analyzing the digital footprints left by potential customers (Lindahl, 2017) to make individual, relevant and meaningful digital communication along the customer journey (Jadli et al., 2022), delivering personalized and automated content under specific rules set by users and marketers, with the goal of attracting, building and maintaining the trust of potential customers (Jäarvinen and Taiminen, 2016).

Marketing automation is empowered and complemented by content marketing, together they make it possible to create automated marketing campaigns in real time (Jadli et al., 2022) with personalized content streams for targeted audiences enabling multiple interactions across the company's various communication channels. B2B content marketing includes images, videos, e-books, guides, podcasts, webinars, infographics, blog texts, social media posts, digital brochures (Jäarvinen and Taiminen, 2016).

The concepts of Marketing Automation, Content Marketing, and a lead scoring system are closely linked (Jäarvinen and Taiminen, 2016) and their efficient integration can contribute to increase the conversion rate (Essi Pöyry and McFarland, 2017), generate high quality Sales leads (Stadlmann and Zehetner, 2022) and delivers a higher ROI for the marketing activities performed (Heimbach et al., 2015).

Although little attention has generally been given to the study of B2B Marketing digitalization (Hofacker et al., 2020), digitalization is an increasingly present topic in the way of doing business in this segment. The use of artificial intelligence technologies driven by machine learning methods and Big Data have a great impact on digital advertising and marketing (Gao et al., 2023), and on the other hand, in terms of lead generation, possibly the greatest contribution of artificial intelligence is the ability to target customers in a highly personalized and individualized way (Syam and Sharma, 2018), for which a deep understanding of consumer behavior is essential.

### 2.3 Consumer behavior theory

Consumer behavior is a valuable source of information for creating lead scoring models, because it allows you to identify patterns and behaviors to assess the level of interest and the probability of conversion. Understanding how potential customers will interact with the brand is essential. The time spent browsing the website, downloading content, interaction on social networks, webinars attended, requests for quotes or demos, etc., are valuable behavioral data that influence the purchase decision process and are relevant attributes for the lead scoring model.

Consumer behavior theory provides a conceptual theoretical framework for analyzing and predicting consumer behavior (Solomon et al., 2012). It is an interdisciplinary field that includes disciplines such as psychology, sociology, social psychology, anthropology, and economics (Solomon et al., 2012; Kotler et al., 2015; Solomon, 2020). The field of study of this theory is based on and the understanding of consumer decisions and the buying process (Solomon, 2020; Morgan and Hunt, 1994).

The term consumer is used to describe the individual consumer, who purchases goods and services for individual consumption, and the organizational consumer, which includes for-profit and nonprofit, public and private organizations that purchase goods and services for use in the production of other products and services (Solomon et al., 2012).

Consumer behavior includes acquisition or pre-consumption, consumption, and post-consumption, which is the disposition of goods and services by buyers (Jacoby, 1978), who may assume different roles during the process of selecting, purchasing, and using the goods (Solomon, 2020). In consumer behavior theory, the analysis of consumer purchasing behavior begins with the identification of consumer characteristics (Dilogini and Shanmugathas, 2017).

Consumer Behavior Theory analyses the psychological factors (personality, motivation, perception, learning and attitudes), social factors (influence of family, friends, social class and interest groups), economic factors (consumer's ability to pay, economic conditions of the environment such as inflation, interest rate, unemployment), cultural factors (subculture, social class and values) that influence the process of selecting products and services.

The key components of consumer theory are: preferences (basic needs and tastes), perception (how consumers perceive brands), learning (how consumers learn from experience), motivation (the drive or reason that motivates purchase), attitudes (feelings toward a brand), social influences (family, friends and peer groups) and culture.

In consumer behavior theory, decision making is the process of selecting an alternative from among available options (Blackwell et al., 2017). According to Kotler, the stages of the purchase decision process are problem or need recognition, information search, evaluation of alternatives, purchase decision, and post-purchase behavior. On the other hand, the stages in the new product adoption process are awareness, interest, evaluation, trial, and adoption (Armstrong, 2009).

The theory of consumer behavior is well known and has been widely accepted since the seventies (Rau and Samiee, 1981), resulting in a large number of theories and models of consumer behavior (Reina Paz and Rodríguez Vargas, 2023) that have been generated over the years, which is a notable achievement. However, according to Jacoby (1978) and Rau and Samiee (1981), the gap between theory and applied practical models has not been bridged.

Numerous studies have influenced the consumer behavior school (Socorro et al., 2018; Blackwell et al., 2017) offering a variety of approaches, perspectives and models of consumer behavior (Prasad and Jha, 2014; Lancaster, 1966). The models that have made significant contributions to the field of consumer behavior are the Nicosia model (Nicosia, 1966), the Howard-Sheth model (Howard and Sheth, 1969), and the Engel-Blackwell-Kollat model (Engel et al., 1978) (Rau and Samiee, 1981; Jacoby, 1978), most of these studies examine the link between external factors and one or more components of the decision-making process (Darley et al., 2010).

The Nicosia model, proposed by Francesco Nicosia, focuses on the decision to purchase a new product, taking into account the consumer's attitude toward the message, the search and evaluation of the product of interest, and the act of purchase and feedback generated (Nicosia, 1966). The positive contributions found in this model are based on the conceptualization of consumer behavior as a decision process and not as the result of a decision process. The main tool used was a system of differential equations in endogenous micro-variables (Sengupta, 1969). This model shows the relationship between stimuli, consumer characteristics, consumer decision process, and consumer responses (Dilogini and Shanmugathas, 2017).

The Howard-Sheth model explains the process of purchasing a product as a system in which the purchasing behavior is a rational and non-random decision that is made within the limits of the consumer's cognitive and learning abilities, and with the restrictions of having limited information. When the behavior is caused by a marketing stimulus to the consumer or his environment, the event or stimulus is the input of the system, the purchase behavior is the output of the system, and the process variables are the internal stages of the consumer decision process such as perception, learning, attitude, and motivation (Haines, 1970).

The Engel-Kollat-Blackwell (EKB) model integrates various facts, internal and external influences that are part of the consumer's path to purchase. This model takes into account perception, learning and motivation. It consists of several stages: problem recognition, search for alternatives, evaluation of alternatives, purchase and consequences (Goyat, 2011; Prasad and Jha, 2014).

The school of consumer behavior has an impact on marketing and business and encourages companies to understand why customers buy, how much they buy, where they buy, when they buy, and how they buy (Solomon et al., 2012). This allows marketers to develop marketing strategies, market segmentation, communication, advertising, promotion, pricing, market research, developing personalized customer experiences, and developing new products that meet consumers' needs and wants.

Improving customer understanding to build more profitable relationships is essential (Hennig-Thurau et al., 2002; Weinstein, 2014). According to Berry (2002), companies must undertake activities and investments aimed at attracting new potential customers and maintaining and improving relationships with existing real customers (Sun, 2009). Relationship marketing, or customer relationship management as this concept is also known, requires commitment and trust (Morgan, 1994). Relationship marketing or one-to-one marketing can increase the value of a company's existing customers by identifying, differentiating, interacting with, and personalizing products and services that best meet their individual needs (Peppers et al., 1999).

Understanding consumer behavior makes it easier to analyze the characteristics of prospects who have become actual customers, which can be used to develop lead scoring models. B2B buyers expect brands to anticipate their needs and provide them with personalized experiences, and customer data is considered a key issue in being able to provide this personalization (Purcarea, 2019).

Knowledge of consumer behavior, market segmentation, and lead scoring models complement each other to enable more effective and customer-centric marketing strategies.

### 2.4 Lead scoring model

A central part of B2B digital marketing is the concept of lead scoring modeling, as it allows organizations to focus their efforts on the most likely and profitable opportunities (Lindahl, 2017). The lead scoring process allows the company through data analysis to predict the weight of each lead according to its probability of conversion (Nygård and Mezei, 2020; Jadli et al., 2023) and was developed by assigning numerical values to each lead generated, and then scoring them based on accumulated points. Points are usually assigned based on the amount of information shared by the lead and based on the lead's involvement with the website or brand of the branded company (Nair and Gupta, 2021).

Lead qualification can be considered a subtask of customer relationship management (CRM) (Nygård and Mezei, 2020). CRM is the methodologies, software and internet capabilities that help a company to manage customer relationships in an organized way (Xu et al., 2002). CRM can include lead management system (LMS) tools to streamline lead generation, management, nurturing, and follow-up. Lead management systems (LMS) are information technology tools that help automate and filter leads and are list-based and queue-based, providing the sales representative with a list of leads filtered according to scoring models, or certain predefined business rules and a given workflow sequence respectively (Ohiomah et al., 2019).

CRM integrates and analyzes data that were generated from formal and informal interactions between customer and supplier (Chatterjee et al., 2021) providing real-time information that can be collaboratively queried by members of a company. The analysis of this data is indispensable for the implementation of successful strategies where artificial intelligence and CRM systems are vital tools in a business environment with decision-making processes that are increasingly data-driven (Saura et al., 2021).

The benefits of establishing a good lead scoring system modeled with information from CRM are evident and allow Marketing teams to reduce the costs and time involved in lead qualification and prioritization (Jadli et al., 2022), to outline efficient marketing strategies and content, which could lead to overall business optimization (Dordevic, 2019).

You can combine attribution models, whether first-click, last-click, multi-touch, or others, to more effectively prioritize prospects. These frameworks improve your understanding of prospect behavior and provide essential input for formulating superior resource allocation tactics.

Without the addition of customer touchpoint information, it's impossible to analyze how these individual interactions drive conversion. For example, first-click attribution models give sole credit to the first interaction, while last-click models reward the last interaction that occurs before conversion (Li et al., 2016; Ben Mrad and Hnich, 2024). Multi-touch attribution, unlike the previous one, considers all touchpoints in the customer journey, providing a holistic view of the customer journey and the customer's relationship with the company (Hülsdau and Teuteberg, 2018; Lee, 2010).

Using these models, companies can assign different weights to each type of interaction to facilitate the scoring of potential customers. Using this approach, scores are modified to better represent the conversion behavior of each prospect (Jadli et al., 2022; Josephine VL et al., 2024; Nygård and Mezei, 2020). Specifically, if a customer interacts multiple times through multiple channels, that customer will receive a higher score and thus have a higher probability of conversion than another customer who receives a lower score because he or she has fewer interactions (Li et al., 2016; Ronen et al., 2021).

In summary, this new system of attribution models and machine learning not only allows for more efficient prioritization of prospects, but also better allocation of resources to achieve higher overall conversion success.

### 2.5 Classification of lead scoring models

Lead scoring models can be classified into two segments; traditional models and predictive models. Traditional lead scoring models use various traditional predictive methods that rely on personal experience, intuition, judgment and cognitive ability of sales and marketing teams (Rohaan et al., 2022; Wu et al., 2024a).

Traditional or manual lead scoring methods are based on rules, points and scorecard models and usually lack formal statistical validation. In these traditional lead scoring models sales reps spend too much time dealing with a large volume of low quality leads that will not convert into customers as they can become inaccurate, arbitrary and biased methods (Nygård and Mezei, 2020; Eitle and Buxmann, 2019; D'Haen et al., 2013). Table 1 shows a traditional lead scoring model, with manual assignment of values to the different characteristics of the leads belonging to the company in the case study.

**Table 1:** Traditional lead scoring model.

**Potential customer interactions with company content**	**Score obtained**
Duration within the website	10
Visited more than one web page (subdomain) per session	5
Downloaded some content from the website	10
Use of CTA	35
Request for a quote	30
Request for a demo/request for more information	10
Incoming call	10
Email marketing campaigns attended	10
2-4 Email marketing campaign openings	2
5 or more Email marketing campaign openings	5
Email response to a campaign	10
Interactions via social networks	10
Facebook/Instagram	5
Youtube/Tik Tok/Linkedin	10
Other interactions	15
Webinars	5
Workshops	5
Number of interactions with the company (touches)	20
1–3 contact interactions	10
More than 4 interactions	10
Date of the last interaction <1 week	10

Predictive models rely on historical data for the identification of relevant patterns and attributes to calculate lead scoring (Wu et al., 2024b). The goal of a learning algorithm is to learn from training data to predict class labels for unanalyzed observations (Tharwat, 2021). Predictive models are heavily supported by data analytics and Big data technologies, which are helpful in modeling the likelihood of lead conversion, and trend detection through predictive analytics of data available to an organization (Nair and Gupta, 2021). These technologies can extract relevant data and transform it into new knowledge that generates value to the company (Figueiredo et al., 2021).

Scoring models can be based on demographic segmentation or behavioral segmentation. The demographic model is oriented to score audiences with standard data such as age, gender, position, industry, location, etc., while the behavioral lead model analyzes the actions performed by potential customers online such as type and number of pages visited, time spent browsing the website, content downloads, etc (Boyer and Hult, 2005). In the behavioral approach we find the engagement-based lead scoring model that suggests that a high engagement with the brand will result in a high conversion, this model analyzes the interactions that potential customers have with the brand (Nair and Gupta, 2021), since through data analysis it is possible to explore the interest of a user, analyzing the interactions made, the sequential behavioral characteristics of the clicks made, all with the aim of increasing the accuracy in predicting the behavior and interests of that user (Gan and Xiao, 2019).

Some authors such as Bondarenko et al. (2019) and Priya (2020) classify leads according to their probability of conversion under a temperature approach considering them cold, warm and hot. Cold leads are those who know nothing about the company and do not seem to have no need for the products and services the company offers, warm leads already know something about the company, have heard about the company, have read articles, follow the company's social networks and are likely to already understand the company's offering and value. Hot prospects are ready to make the purchase and payment for the products (Bondarenko et al., 2019; Priya, 2020).

Often companies combine the mixed approach based on demographic and behavioral data for the creation of traditional or predictive mode lead scoring models.

### 2.6 Other work related to lead scoring models and their applicability across industries and regions

Predictive lead scoring models are not only indispensable, they are being implemented in a variety of industries. Information technology (IT)-related companies are at the forefront of implementing predictive lead scoring models and automating routine processes. Lead scoring models can be found in various disciplines, such as banking (Djurisic et al., 2020), science (Gouveia and Costa, 2022), services (Bohanec et al., 2016), logistics (Pereira, 2021) and packaging (Mortensen et al., 2019), telecommunications (Espadinha-Cruz et al., 2021), among others, demonstrating their remarkable versatility and importance in the industry of different sectors in Europe, Asia and the Americas.

The implementation of lead scoring models depends on the availability of accurate data on leads and sales in an organization, since the study of such information facilitates their classification and prioritization, regardless of the industry and country in which they operate. End-to-end optimization of the organization can be achieved by implementing machine learning techniques in scoring models. These techniques enable the application of a personalized model for customers, a capability that traditional scoring systems of competitors undoubtedly cannot achieve (Dordevic, 2019).

As part of the literature review, other existing studies related to the construction of potential customer scoring models were found in various industries and geographic regions, which are discussed below.

Authors such as Monat (2011) present qualitative lead scoring studies focused on lead characterization. D'Haen et al. (2013) developed a model that sought to generate a ranked lead list with high predisposition to conversion, based on information from the company's current customers and using the nearest neighbor algorithm to obtain predictions, as well as logistic regression, decision trees and neural networks. They considered their findings positive as the model increased the conversion rate of potential customers (D'Haen et al., 2013).

Other studies based on multinomial logistic regression analysis explained how the complexity of the sales situation affects lead performance in B2B environments, predicting that increased complexity and collaboration in the sales situation causes more lead opportunities to be lost or canceled. They did this study using mixed methods, with data originating from sales force interviews and lead data recorded in an IT company's CRM, where they categorized leads as won, lost, and canceled. Their data analysis technique categorized leads into won, lost and canceled. The data was collected through interviews with sales personnel and extracted directly from the CRM of a global IT company (Virtanen et al., 2015).

Gabryel et al. (2018) conducted studies on lead qualification models in a Polish bank using simple calculations performed by the Bag-of-words algorithm. The research was conducted by taking data collected from their web forms. The classical bag-of-words algorithm is based on the concept of text search methods within document collection. Negatively scored opportunities were moved to the end of the opportunity queue to prioritize the most important opportunities. This scoring model discriminated forms filled out by human or real leads from forms completed by bots (Gabryel et al., 2018).

Eitle and Buxmann (2019) proposed a study integrating business analytics in the form of machine learning involving lead management where they demonstrated that CatBoost and random forest tools have high predictive performance in lead and opportunity management due to their good handling of categorical big data. In their 2019 study, Mortensen et al. presented a case study that employed a predictive approach to identify opportunities with a higher propensity to be won in a paper and packaging company (Mortensen et al., 2019). The researchers used several techniques, including binomial logit, decision trees, and random forest, to demonstrate an improvement in the accuracy of opportunity prediction.

In Priya (2020), the authors developed a traditional lead scoring model and scored leads according to their likelihood of conversion into hot, warm, or cold leads. This model was executed with unstructured data in a simple spreadsheet, without the use of technology tools. The marketing team was required to utilize their expertise to define the ideal customers and the parameters that influenced purchases, assigning scores from –2 to +10 to each parameter. The marketing team was tasked with identifying three types of decision parameters: characteristic data, behavioral data, and negative data. The lead value was determined as the sum of each lead's score and the comparison of each lead's score to the total score. The researchers posited that the limitations of traditional lead scoring models could be overcome by emphasizing negative data in the model. This study was conducted in India.

Nygård and Mezei (2020) presented in their study the feasibility of using machine learning as an alternative to the manual customer scoring processes that are still widely used. They found that there are major challenges in preparing and preprocessing lead activity data and found that while the random forest model performed well there were opportunities to improve the models by extending the optimization of the data parameters.

**Figure 1:**
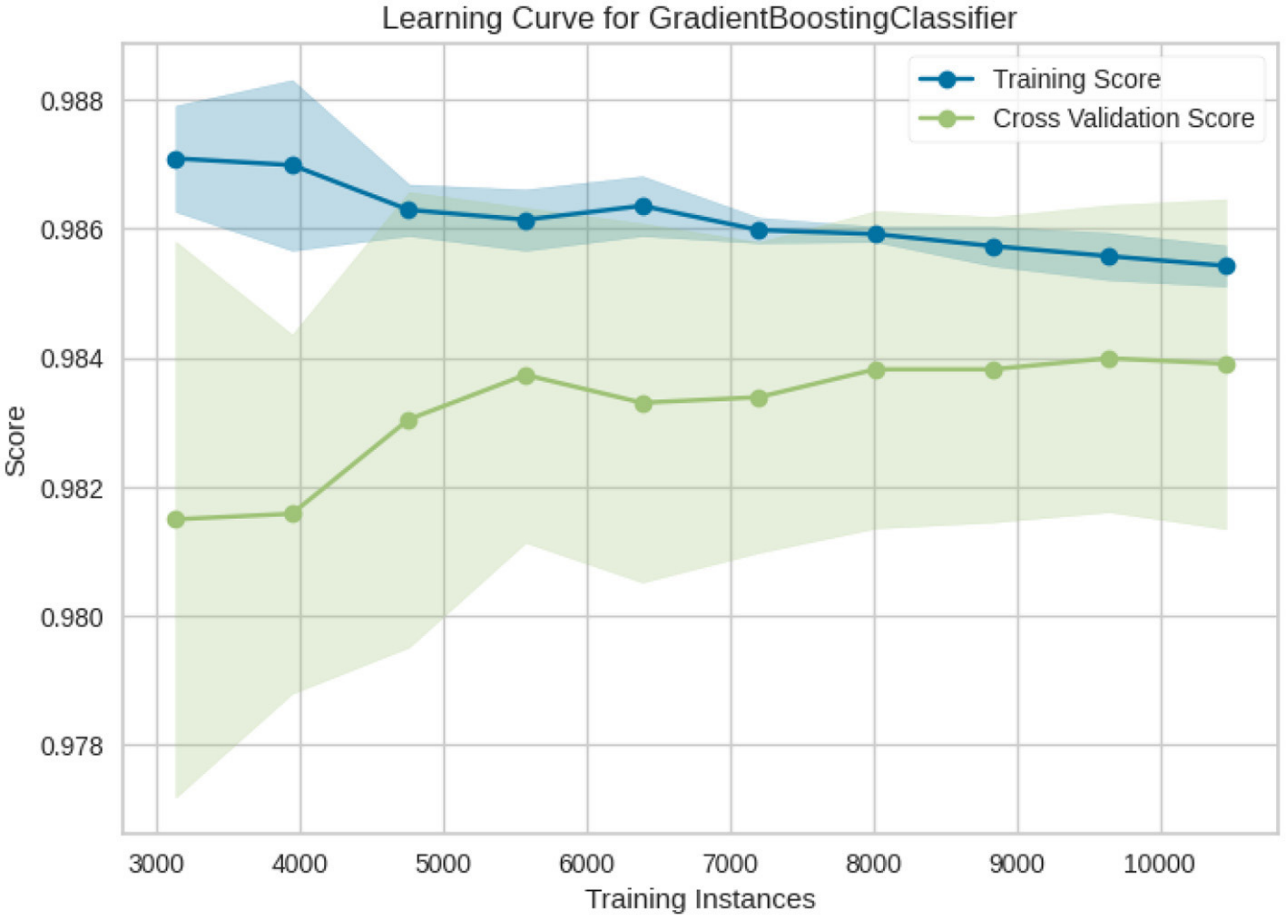
Learning curve of Gradient boosting classifier.

In their study, Djurisic et al. (2020) present predictive models used to classify and segment credit card users with the aim of improving revenue and reducing expenses (Figure 1). Concurrently, Pereira (2021) proposed a decision tree model augmented by the CART algorithm, with the objective of predicting the conversion of potential customers in a logistics company.

Espadinha-Cruz et al. (2021) proposed a method to estimate the probability of lead conversion in a telecommunications company in Portugal. Their predictive model suggests the application of data mining for the optimization of lead and opportunity management processes.

Stadlmann and Zehetner (2022) compared AI-based methods with traditional methods, finding that data mining tools may be even weaker than traditional methods if web tracking activities are influenced by the underlying databases. They find it advisable not to rely on a single approach and suggest combining traditional and AI methods.

In the work, Gouveia and Costa (2022) developed a model for predicting leads in the education sector in Brazil, applying logistic regression and finding significant improvements in the conversion rate. It was helpful in the customer segmentation process and provided time savings for the work teams involved in filtering potential customers.

Jadli et al. (2023) investigated the advantages of using machine learning algorithms to optimize lead scoring as a replacement for traditional lead scoring systems. Their experiment analyzed the probability of conversion based on the demographic and behavioral data of website visitors. They employed various machine learning techniques and found that the random forest model outperformed the others. In another study on lead scoring, Jadli et al. (2022) concluded that artificial intelligence-based scoring models are effective in identifying high-quality leads among website visitors, resulting in a better ROI for companies.

In Wu et al. (2024b), the authors in their study “The state of lead scoring models and their impact on sales performance,” reviewed 44 studies of lead scoring models published between 2005 and 2022 and found that the use of lead scoring models has a positive relationship with the following assumptions; Lead scoring models improve lead conversion rate, reduce costs involved in lead conversion, increase revenue and profit and increase the number of high quality leads. They concluded that artificial intelligence-based predictive lead scoring models positively influence sales performance.

Artificial intelligence has the ability to develop and apply predictive algorithms to assist in the creation of lead scoring models. Artificial intelligence systems can analyze past prospect data and determine which actual online and offline features are most likely to generate conversions (Syam and Sharma, 2018).

## 3 Artificial intelligence and its influence on B2B lead scoring models

### 3.1 Introduction to artificial intelligence

Artificial intelligence (AI) can be conceptualized as the use of computational machinery to emulate capabilities inherent in humans and can become designed to have multiple intelligences to perform physical, thinking, and feeling tasks as humans have (Huang and Rust, 2021).

The first studies on artificial intelligence emerged after the Second World War in 1940 with the appearance of the first computer and the work of Alan Turing proposed in 1950 called the Turing Test (Turing, 1950) in which he presented a way to validate whether the computer could have some kind of human intelligence (Prieto and Braga, 2021). Turing called this test the imitation game (French, 2000). Subsequently, McCarthy and a team of researchers at the University of Dartmouth in 1955 (Prieto and Braga, 2021) went deeper into the research on artificial intelligence.

The proposal of artificial intelligence is to convert large amounts of data into meaningful information for the creation and management of superior knowledge in B2B sales, which could come to significantly alter the B2B funnel and its sales stages (Paschen et al., 2019).

In the field of marketing, AI contributes to solving different problems, such as automation of repetitive tasks through mechanical AI, personalization activities with thought AI, and those related to the analysis of human interactions through sentiment AI (Huang and Rust, 2021). Generative AI is also on the rise as it is able to generate seemingly new content such as text, images and audio from training data such as GPT-4 and Copilot (Feuerriegel et al., 2024).

AI with its data mining is useful for extracting meaningful knowledge from large datasets by applying machine learning methods and since it is an iterative process it allows to continue adding new knowledge to the dataset (Aggarwal, 2014). In machine learning machines perform their work through the use of intelligent software which has its basis in statistical learning methods (Kumar et al., 2020). Data mining, inference and prediction as elements of statistical learning are of utmost importance in addressing various real-world problems (Hastie et al., 2009).

### 3.2 Machine learning

We will address machine learning because it is the basis of the functionalities offered by artificial intelligence (Campbell et al., 2020). Machine learning is a branch of artificial intelligence that provides systems with the ability to learn and improve automatically from data without being explicitly programmed, because its algorithms detect patterns and learn to make predictions and recommendations by processing data from previous experiences (Campbell et al., 2020). Machine learning can be broadly classified into supervised learning, unsupervised learning and reinforcement learning.

In supervised machine learning, structured and labeled data is analyzed seeking to predict an outcome and unsupervised machine learning seeks to determine the hidden structure or patterns of an unstructured and sometimes unlabeled data set (Syam and Sharma, 2018). Reinforcement learning is used in situations where there is no dataset, the algorithm will learn by performing different actions and evaluating their success or failure and the immediate and continuous feedback will allow the system to learn while creating a dataset (Campbell et al., 2020).

In supervised machine learning techniques, one starts by collecting relevant labeled data reflecting the characteristics of potential customers, then cleaning the collected data and then dividing it into a ratio of 80/20 or 70/30 to be used for training and testing respectively. The goal of a learning algorithm is to learn from the training data to predict class labels for unanalyzed observations (Tharwat, 2021). After training the model, the results are evaluated and the best performing algorithm is selected to apply to future cases (Rohaan et al., 2022).

The most common predictive supervised machine learning models are regression and classification models (Wu et al., 2024b). Regression models are less common due to the categorical results of lead scoring. These models use linear regression, exponential regression, seasonal ARIMA time series model, and neural networks to forecast lead conversion rates and estimate sales revenue. Classification methods have been applied in many fields of science (Tharwat, 2021) and play an important role in business decision making (Kiang, 2003). One of the areas where they are recurrent is the development of scoring systems and we will address them in a little more detail below.

### 3.3 Predictive ranking models for scoring potential customers

Predictive models based on ranking algorithms are the most requested in the creation of lead scoring models because of their predictive efficiency whenever historical data is available. Classification methods predict qualitative responses by analyzing qualitative variables that are classified as categories or classes. Often the methods used for classification first predict the probability that the observation belongs to each of the categories of a qualitative variable as a basis for performing the classification (James et al., 2023).

Some of the most popular classification algorithms are decision trees, logistic regression, random forest, support vector machines (SVM), K-nearest neighbors, gradient boosted trees, neural networks, Gradient Boosting Machines (GBM) and Bayesian networks (Wu et al., 2024b; Syam and Sharma, 2018). We will address some of them due to the fact that they play a relevant role in the study of this article. Decision trees, this technique is used to iteratively segment the data into subsets of similar characteristics and each leaf of the tree is assigned a class (Aggarwal, 2014). This technique is used to identify factors that convert potential customers into actual customers. It automates prediction using rules extracted from data patterns. Examples include models that optimize sales productivity and automated lead classification systems (Wu et al., 2024a).

Random forest: This technique is based on decision trees and assembles multiple decision trees by combining their predictions to improve their accuracy. It is a suitable technique for classifying prospects with social network data and explanatory models. Despite being considered a black box algorithm, it offers excellent accuracy in lead classification.

Logistic regression is a simple but very effective binary linear classification algorithm (Jadli et al., 2022) that works by mapping a vector of latent features to a range of values of 0,1 using a sigmoid function. It is useful for identifying, qualifying and prioritizing leads as it helps to predict the conversion probability of a lead based on its characteristics and previous purchases.

The k-NN is a machine learning algorithm that takes the “k” nearest neighbors or nearest data to a given query point with missing data and imputes them based on the non-missing values in the neighbors. The nearest and most similar neighbors are found by decreasing a distance function. With this distance calculation it will determine the proximity to make classifications and predictions about the clustering of an individual point (Filipe, 2020) since an observation will be assigned to the group to which most of its K-nearest neighbors belong (Kiang, 2003).

Naive Bayes, is a simple probabilistic classifier that uses probabilities according to Bayes' theorem relating conditional and marginal probabilities of events A and B (Bole and Papa, 2011). This algorithm focuses on building probabilistic models to estimate the probabilities that potential customers belong to target classes (Wu et al., 2024a).

Neural networks simulate the human brain with a large number of interconnected nodes that unlike humans are able to extract information and patterns from messy and complex data. They work by breaking problems into smaller components and solving them iteratively taking into consideration findings from previous stages (Campbell et al., 2020). Neural networks can address unbalanced class labels, reveal typical purchase patterns, and can be implemented in lead scoring models to estimate conversion probabilities (Wu et al., 2024a).

Gradient Boosting Classifier, is an algorithm that uses the “boosting” approach producing highly robust and interpretable competitive procedures for classification (Hastie et al., 2009).

The extreme gradient boosting algorithm (XGBoost) is a supervised learning technique that is based on gradient boosted decision trees (GBDT), combining predictions to make predictions by weighted averaging. This algorithm offers high performance compared to the Gradient Boosting algorithm.

Support Vector Machine (SVM) this technique can make nonlinear classifications efficiently through Kernels, it only requires a subset of the input data, called support vectors to determine the maximum margin that makes the classification (Syam and Sharma, 2018).

Supervised learning algorithms for classification can solve problems related to customer scoring, as they enable artificial intelligence analysis of the data by structuring and classifying it in such a way that it provides greater insight into the customer and their preferences (Paschen et al., 2019).

Lead scoring is considered a critical task in marketing departments as it brings effectiveness to Marketing campaigns and will help the sales team to increase the conversion rate (Priya, 2020).

## 4 Methodology

The methodology used was a case study of a B2B SME company providing software for product design and manufacturing for the development of a lead scoring system based on supervised machine learning for the qualification and prioritization of potential customers, since this study had hypothesized that the implementation of the lead scoring model based on machine learning facilitates the prioritization of potential customers compared to traditional models in B2B markets.

Several supervised machine learning classification techniques were used to estimate the conversion probability of B2B leads. Classification techniques were selected in this study because of their suitability for predicting responses to qualitative variables, as they can predict the category or class to which a new observation belongs based on previous examples, thus the classification algorithms can predict which leads might eventually purchase based on information from customers who previously purchased from the company.

The company in this study was facing difficulties in predicting and prioritizing its leads even though it had traditional lead scoring model, it maintained a strong interest in improving the process and assertiveness of lead scoring activities in order to increase its lead conversion rate.

**Figure 2:**
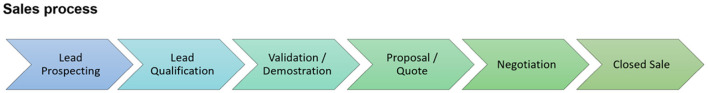
Sales process of the case study company.

Its consultative sales process consists of several stages that can be seen in Figure 2, in its business activities it generates many leads to qualify, however its lead conversion rate is very low, due to the fact that many times sales representatives delay in attending leads that will convert because they are spending time in vain looking to convert other leads that are not ready to convert, eventually sales representatives lose interest in calling the rest of the leads or the leads will look for a quick response with the competition, causing the loss of resources invested in demand generation.

The research proposed a solution to the need for automatic lead qualification and demonstrates the relevance of applying artificial intelligence to this type of problem.

## 5 Lead prediction model development process

**Figure 3:**
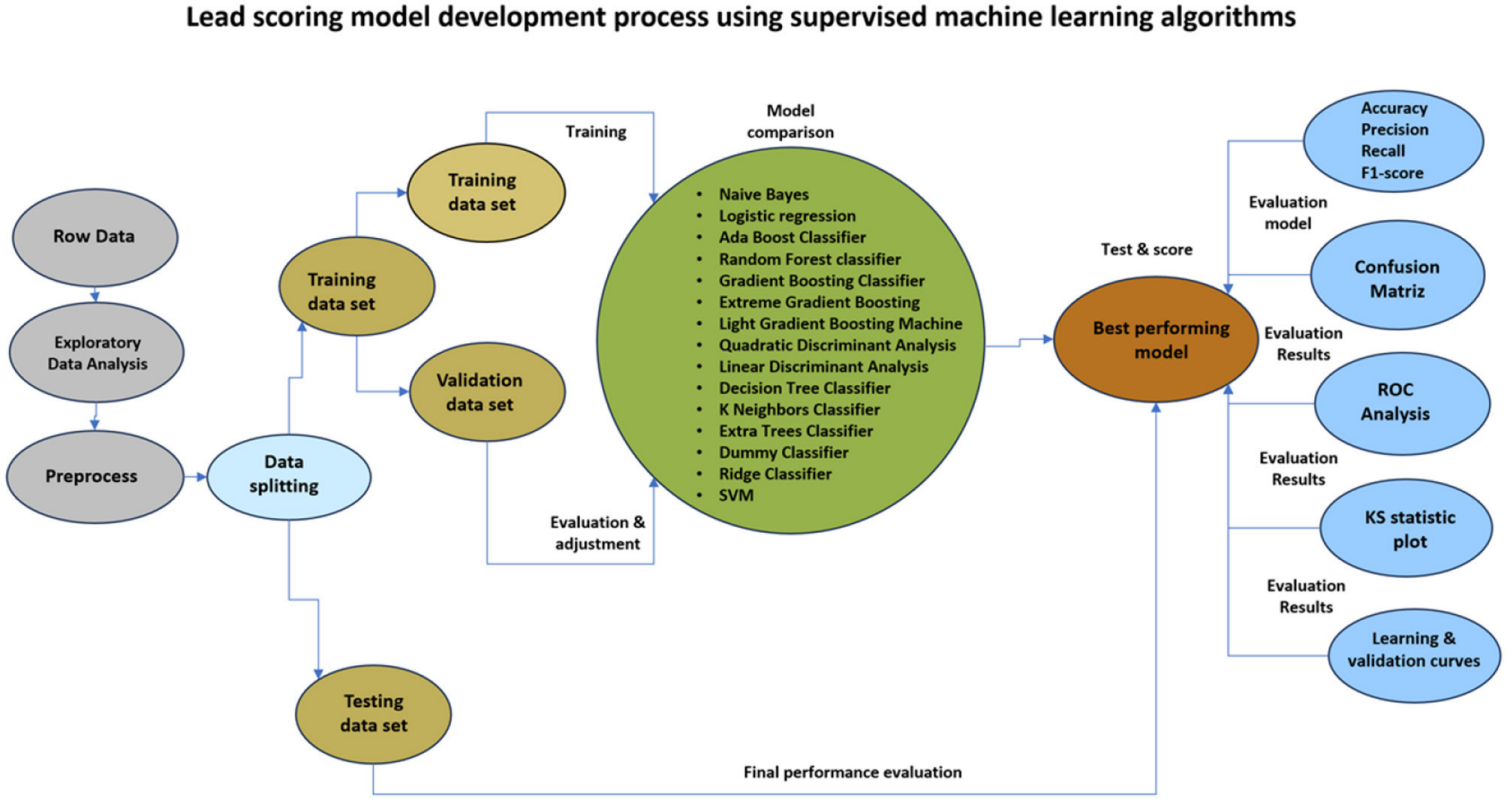
Lead scoring model process using supervised machine learning algorithms.

In general terms, the process used to develop the customer prediction model began with the collection of historical data of potential customer records of the company from May 2020 to April 2024. The data was then post-processed to make it suitable for analysis. Afterwards, the data was divided in a ratio of 70/30 for training and testing respectively. Several classification algorithms were used to train the models. Subsequently, the performance of the machine learning models was evaluated by applying the metrics of Accuracy, precision, recall, f1-score, confusion matrix and ROC analysis. Figure 3 breaks down the process used for the development of the lead scoring model under the supervised machine learning approach.

### 5.1 Data description

Real business data considered relevant were extracted from the company's CRM Microsoft Dynamics customer relationship management system for exploration and understanding. The dataset contained information related to leads and transactions conducted with the company from January 2020 to April 2024. The structure of the dataset consisted of 23,154 records and 67 fields. The dataset had numeric, categorical and text data and contained fields such as lead ID, lead classification, source of origin (specific source of the lead), main contact, telephone, mail, status (location), state (status), reason for status (status), date of last activity, among other data. In the data dictionary, each field was defined, the type of value it was, and a short description was made (see Supplementary Appendix 5).

### 5.2 Data processing

#### 5.2.1 Exploratory data analysis

To guarantee the transparency and reproducibility of the study, a detailed analysis of the data used in the development of the classification model has been included. This analysis includes the distribution of key variables through histograms and other graphical representations that allow for the identification of possible patterns and outliers. Given the large volume of variables analyzed, the individual histograms are presented in the Supplementary Appendix 10, which allows for detailed consultation without overloading the main body of the text.

**Figure 4:**
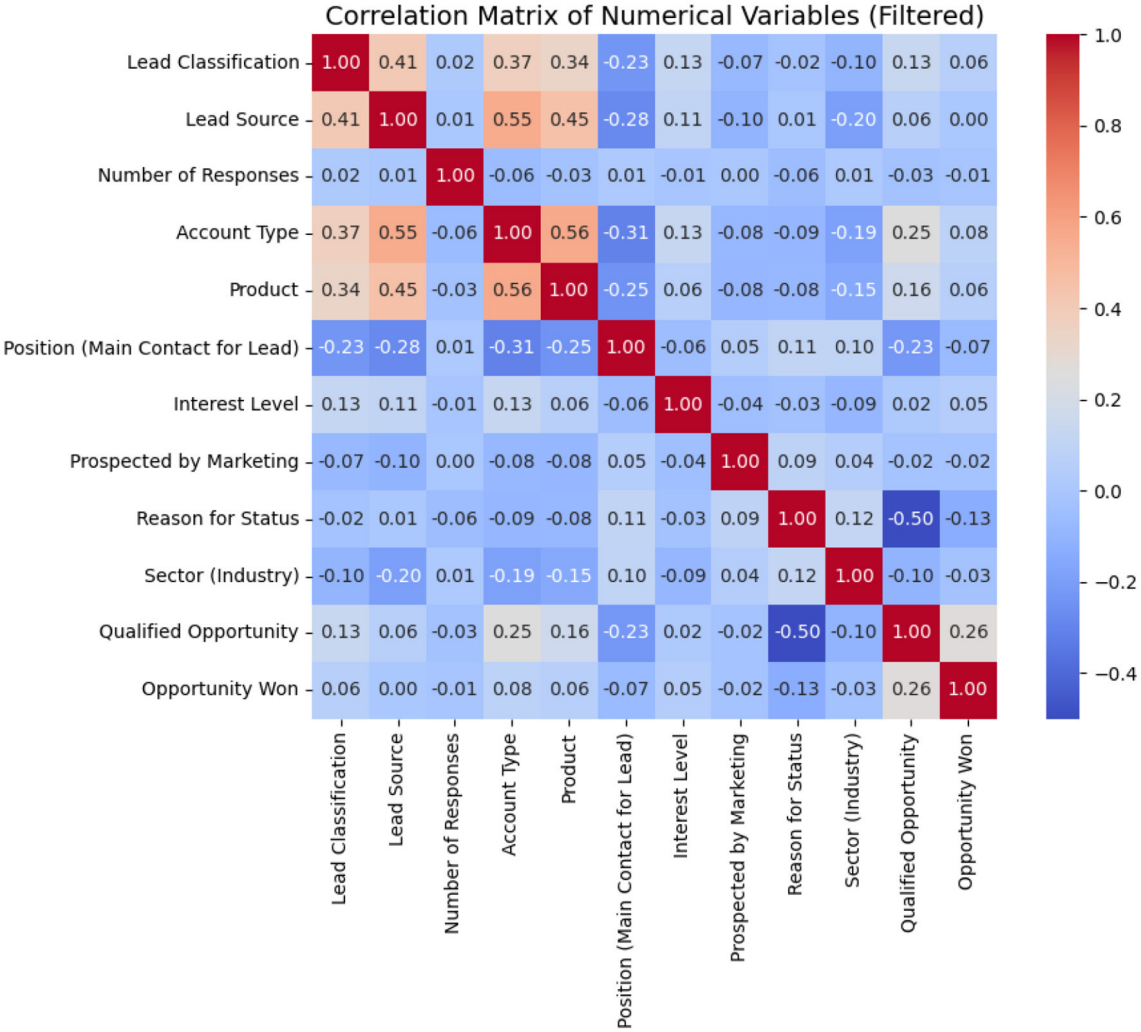
Correlation matrix.

Figure 4 shows the correlation matrix of the numerical variables used in the study. It is important to point out that this matrix has been filtered due to the presence of missing data in some variables, which led to their exclusion to ensure a more accurate representation of the relationships between the available data.

The following key points can be highlighted:

- Strong relationships between variables: A significant positive correlation is observed between Account Type and Lead Source (0.55), as well as between Product and Account Type (0.56), suggesting that certain types of accounts are more associated with specific sources of leads and products.
- Moderate correlations with lead classification: The Lead Classification variable shows a moderate correlation with Lead Source (0.41) and Account Type (0.37), indicating that the type of account and the source of the lead can influence its classification.
- Impact of variables on opportunity conversion: The Opportunity Won variable does not present particularly high correlations with any variable in this matrix, which suggests that other non-numerical factors may play a crucial role in the conversion of opportunities.
- Effect of negative values: Some variables show negative correlations, such as Sector (Industry) and Reason for Status (–0.50), which could indicate that certain sectors have specific reasons for the classification of their leads.

**Figure 5:**
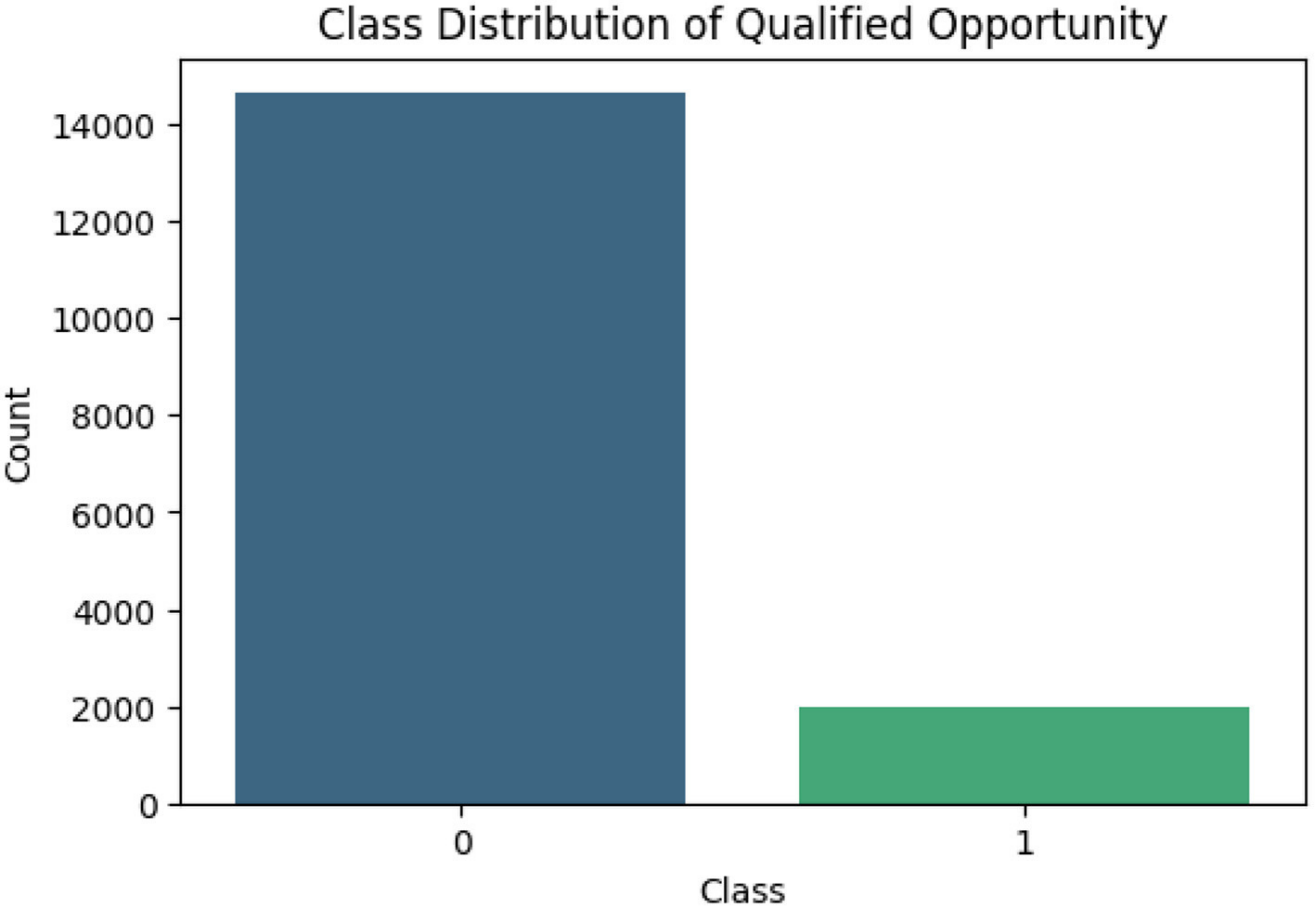
Class distribution of qualified opportunity.

The “Qualified Opportunity” variable is binary and highly unbalanced, see Figure 5 for the distribution of classes. Class 0 (unqualified opportunity) represents the majority of observations, while class 1 (qualified opportunity) is represented by a small number of records. With fewer examples in the minority, models trained on this data may be less effective at predicting classifications in this group. Algorithms tend to favor the dominant class, resulting in lower performance in identifying qualified opportunities.

Tukey's rule for detection of outliers in a data set should be applied. It takes into account *Q*1 and *Q*3 and interquartile distance (IQR) and categorizes the data value(s) that lie outside the range: *Q*1 − 1.5 × *IQR* and *Q*3 + 1.5 × *IQR* as outliers. Thus, this rule is applied to the data to check its extreme values and that the data being processed are consistent and representative (see Table 2). This method enhances analytical capability by ensuring data integrity and consistency before any calculation or analysis.

**Table 2:** Analysis of outlier detection in the Dataset.

**Variable**	**Outlier percentage**	**Recommendation**
Lead classification	0.00%	Manageable: consider transformations or removal
Lead source	13.26%	Moderate: investigate causes and handle appropriately
# of Responses	5.01%	Moderate: investigate causes and handle appropriately
AccountType	0.00%	Manageable: consider transformations or removal
Product	4.95%	Manageable: consider transformations or removal
Email	0.00%	Manageable: consider transformations or removal
Work phone	0.00%	Manageable: consider transformations or removal
Title (Primary Contact for Lead) (Contact)	18.80%	High: review data quality or collection processes
Do not allow email	0.00%	Manageable: consider transformations or removal
Do not allow bulk email	0.00%	Manageable: consider transformations or removal
Do not allow faxes	0.00%	Manageable: consider transformations or removal
Do not allow phone calls	0.00%	manageable: consider transformations or removal
Are you the decision maker?	0.00%	Manageable: consider transformations or removal
Sales stage	0.00%	Manageable: consider transformations or removal
I speak to the CEO	0.00%	Manageable: consider transformations or removal
Marketing materials	0.00%	manageable: consider transformations or removal
Interest level	3.75%	manageable: consider transformations or removal
Prospected by marketing	6.10%	moderate: investigate causes and handle appropriately
Reason for status	0.00%	Manageable: consider transformations or removal
Industry (industry)	0.00%	Manageable: consider transformations or removal
Qualified opportunity	11.84%	moderate: investigate causes and handle appropriately
Won opportunity	0.92%	Manageable: consider transformations or removal

Most of the numeric variables include a manageable instance of outliers. This signal indicates that the overall data is found good and needs a small transformation to be used more effectively. However, there are some important variables that need further assessment. In this regard, Lead Source (13.26%), Prospected by Marketing (6.10%), and Qualified Opportunity (11.84%) are listed as outliers. These percentages may stem from a variety of reasons, and further investigation is warranted. These might stem from capture errors, random variations by data entry people, or data-specific properties which we need to adjust for. These are all factors associated with the lead source and its quality, which signifies proper handling for correct insights.

On the other hand, Title (primary contact for the lead) reaches 18.80 % of outliers, which could also point to data quality issues. This is an extremely important variable from a marketing perspective as it tells us who is the main contact in the organization. For marketers, it is important to obtain this information so they can create personalized strategies, focus on a small number of customers, and make the most of the data and resources they work with. Its high percentage indicates that it must be removed. Doing so would restrict the data that could be used to derive insights, and the variable should be kept.

### 5.3 Data cleaning

With the help of PyCaret, data cleaning was performed, identifying, correcting and removing errors, inconsistencies and duplicate data from the dataset in order to ensure the accuracy and reliability of the results. PyCaret a Python library that facilitates data preparation for model training (Ali, 2019), provides functions for data cleaning, outlier handling, missing value imputation and coding of categorical variables. Fields were selected based on their impact on a potential customer's probability of purchase and records were selected based on the amount of information contained. Some columns were excluded from the database because they were created at different times, which meant that only some records had values and were therefore eliminated. At this stage the database structure was reduced to 16,600 records and was reduced to 22 fields.

### 5.4 Coding of categorical variables

In some cases it was necessary to code categorical variables using techniques such as one-hot encoding and label encoding in the following fields:

- Level of interest contained four categories and we proceeded to use label encoding to assign them a numerical value prioritizing the level of greatest interest.
- Purchase period, it was scaled between the highest and lowest purchase period and was given a value of 1–5, to denote its importance the options of immediate and 3 months were given a value of 1 and the least important option was given a value of 5.
- Status reflecting the status of the potential client was expressed in terms of open, won and lost and was changed to a numerical value.
- Some text categories were transformed to bilevel values, e.g., qualified opportunity to determine which customers had been qualified and which had not an opportunity to determine whether or not the lead had completed the purchase.

The PyCaret classification module was used to solve classification problems in the database columns. This model predicts the possibility of generating new categorical variables to be taken from the input values and the columns were increased from 45 to 22, leaving the final structure of the dataset with 16,600 records and 22 fields.

### 5.5 Division of the data set

After data processing, the dataset was left with 16,600 records, which was randomly divided leaving 70% for training the algorithms and the other 30% for testing. The training dataset was in turn divided into 10 parts for better results.

### 5.6 Training and comparison of models

In order to perform an accurate training and comparison of models, the field “qualified opportunity” was defined as a target variable. Since PyCaret allows to compare multiple machine learning models in this study the data were analyzed through several classification algorithms such as Naive Bayes, Logistic regression, Ada Boost Classifier, Random Forest classifier, Gradient Boosting Classifier, Extreme Gradient Boosting, Light Gradient Boosting, Machine, Quadratic Discriminant Analysis, Linear Discriminant Analysis, Decision Tree Classifier, K Neighbors Classifier, Extra Trees Classifier, Dummy Classifier, Ridge Classifier and SVM.

### 5.7 Evaluation of model performance

Some of the evaluation metrics applied to examine model performance are described below:

- Accuracy is the proportion of correctly predicted sales over the total number of sales predictions (correct or wrong).
- Precision is the proportion of true positives to the total number of positive predictions.
- Recall (sensitivity or completeness) is the proportion of true positives to the total number of true positives.
- F1 score which represents the harmonic mean of precision and recall. It is the percentage of sales that were correctly predicted out of the total number of actual sales predicted (Rohaan et al., 2022). Its value range is from 0 to 1, and high values in this metric indicate high classification performance (Tharwat, 2021; Wu et al., 2024).

In order to evaluate the results of the best models, the following tools were used:

- Confusion matrix: A confusion matrix summarizes the classification job performance of a classifier with respect to some test data.
- Receiver Operating Characteristics (ROC) is used to evaluate many systems, including machine learning systems. This two-dimensional plot is used to make a trade-off between the benefits that would be true positives and the costs that would be false positives (Tharwat, 2021).
- Area under the ROC curve (Receiver Operating Characteristics AUC) expresses the extent to which the prediction model can distinguish between classes. This metric takes values in the range of 0 to 1. Any value below 0.5 indicates that the classifier is unrealistic and that the prediction model cannot differentiate classes. A value of 1 indicates that the model works correctly to differentiate between classes.

### 5.8 Model evaluation metrics

The overall result of the fifteen different models is presented in Table 3. Gradient Boosting Classifier: Has the highest accuracy (0.9839), along with high AUC (0.9891), recall (0.9586) and MCC (0.9252), indicating excellent overall performance on all metrics. LightGBM: It has very similar performance to Gradient Boosting Classifier, with slightly lower recall and precision, but very close values in all metrics. Ada Boost Classifier: Excels in AUC (0.9891), suggesting an excellent ability to distinguish between classes, but has a slightly lower performance in terms of precision and F1 compared to the previous models. Ridge Classifier: It has a very high accuracy (0.9605) in terms of precision, but a very low recall (0.1759), indicating that it detects few true positives, but when it does, they are very accurate.

**Table 3:** Evaluation metrics of the applied models.

**Model**	**Accuracy**	**AUC**	**Recall**	**Prec**.	**F1**	**Kappa**	**MCC**
Gradient boosting classifier	0.9839	0.9891	0.9586	0.9106	0.9338	0.9247	0.9252
Light gradient boosting machine	0.9835	0.9885	0.9535	0.9112	0.9318	0.9224	0.9228
Extreme gradient boosting	0.9821	0.9872	0.936	0.9149	0.9248	0.915	0.9152
Logistic regression	0.9818	0.9775	0.9462	0.9047	0.9248	0.9144	0.9144
Random forest classifier	0.9803	0.9817	0.9317	0.9048	0.9179	0.9067	0.9069
Ada boost classifier	0.9799	0.9891	0.9375	0.9057	0.9169	0.9055	0.9058
Extra trees classifier	0.9779	0.9735	0.9091	0.9091	0.9091	0.8941	0.8942
Decision tree classifier	0.9725	0.9455	0.875	0.8905	0.8823	0.8667	0.8677
K neighbors classifier	0.9377	0.931	0.654	0.7839	0.7127	0.6781	0.6819
SVM - linear kernel	0.9332	0.9753	0.6671	0.8331	0.8331	0.6393	0.6784
Linear discriminant analysis	0.9271	0.9598	0.4913	0.8217	0.6143	0.5769	0.5679
Ridge classifier	0.9015	0.9598	0.1759	0.9605	0.2964	0.2697	0.3678
Naive Bayes	0.8971	0.9361	0.2188	0.7149	0.3344	0.2954	0.3575
Quadratic discriminant analysis	0.8816	0.9351	0.2149	0.7149	0.3314	0.2852	0.3575
Dummy classifier	0.8816	0.5	0	0	0	0	0

Values highlighted in yellow represent averages of key metrics, making it easier to compare performance across models in different classification scenarios.

### 5.9 Best performing models

After performing multiple tests with different training and validation samples to ensure the integrity of the results, it was found that the best models for this dataset were Gradient Boosting Classifier, Light Gradient Boosting Machine, Extreme Gradient Boosting and Logistic regression. Table 4 shows that the Gradient Boosting Classifier model has a superior performance with respect to the other classifiers.

**Table 4:** Best performing models for this study.

**Model**	**Accuracy**	**AUC**	**Recall**	**Precision**	**F1**	**Kappa**	**MCC**	**TT (Sec)**
Gradient boosting classifier	0.9839	0.9891	0.9586	0.9106	0.9338	0.9247	0.9252	0.903
LightGBM	0.9835	0.9885	0.9535	0.9112	0.9318	0.9224	0.9228	0.393
XGBoost	0.9821	0.9872	0.936	0.9149	0.9252	0.915	0.9152	0.191
Logistic regression	0.9818	0.9775	0.9462	0.9047	0.9248	0.9144	0.9148	0.247

Values highlighted in yellow represent averages of key metrics, making it easier to compare performance across models in different classification scenarios.

**Figure 6:**
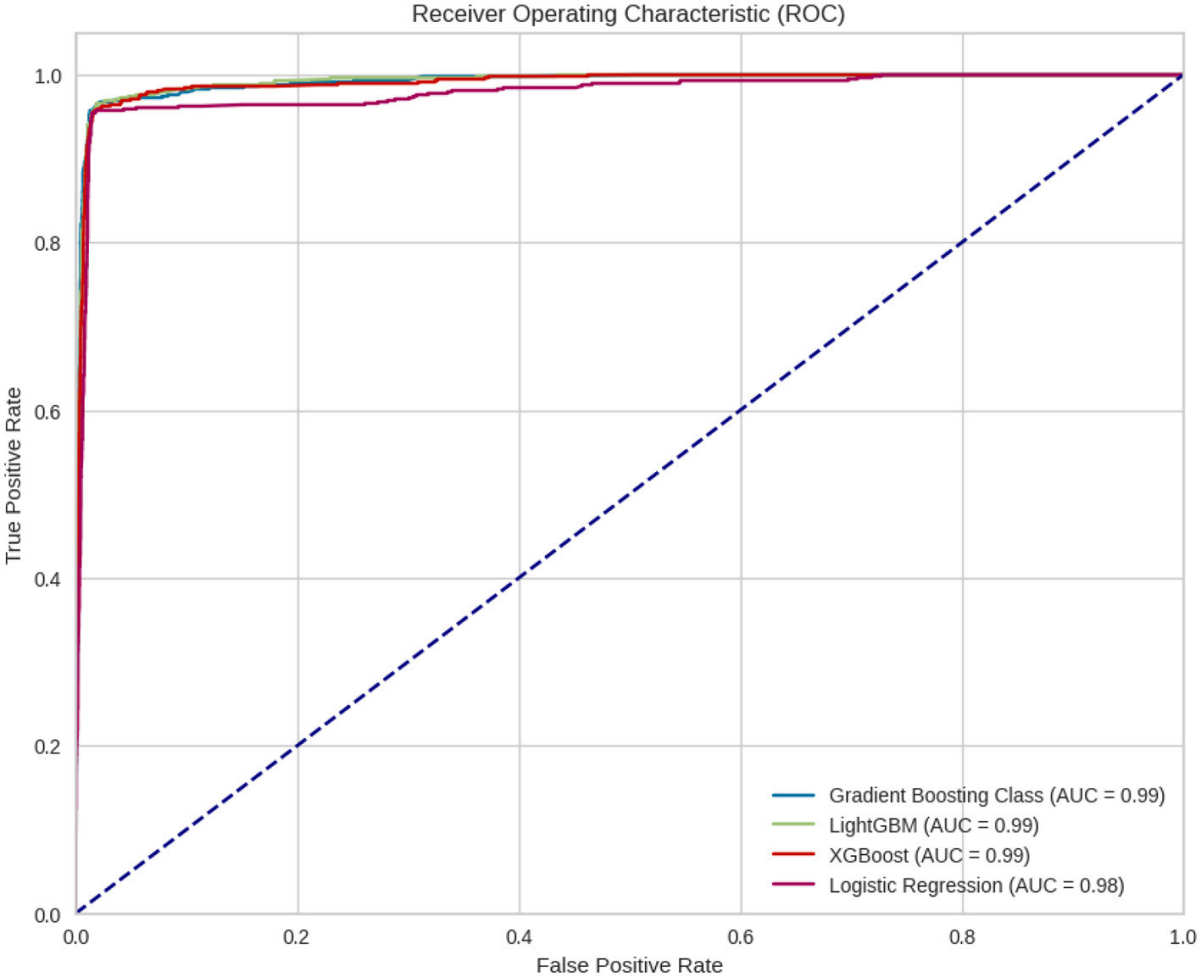
ROC curve for the four different models.

Below in the Figure 6 is the ROC curve where the four different models are compared. It can be observed that all four models have very high AUCs, with Gradient Boosting, XGBoost and LightGBM showing an AUC of 0.99, indicating that they are very good classifiers and slightly better than logistic regression.

### 5.10 Evaluation metrics of the best performing models

**Figure 7:**
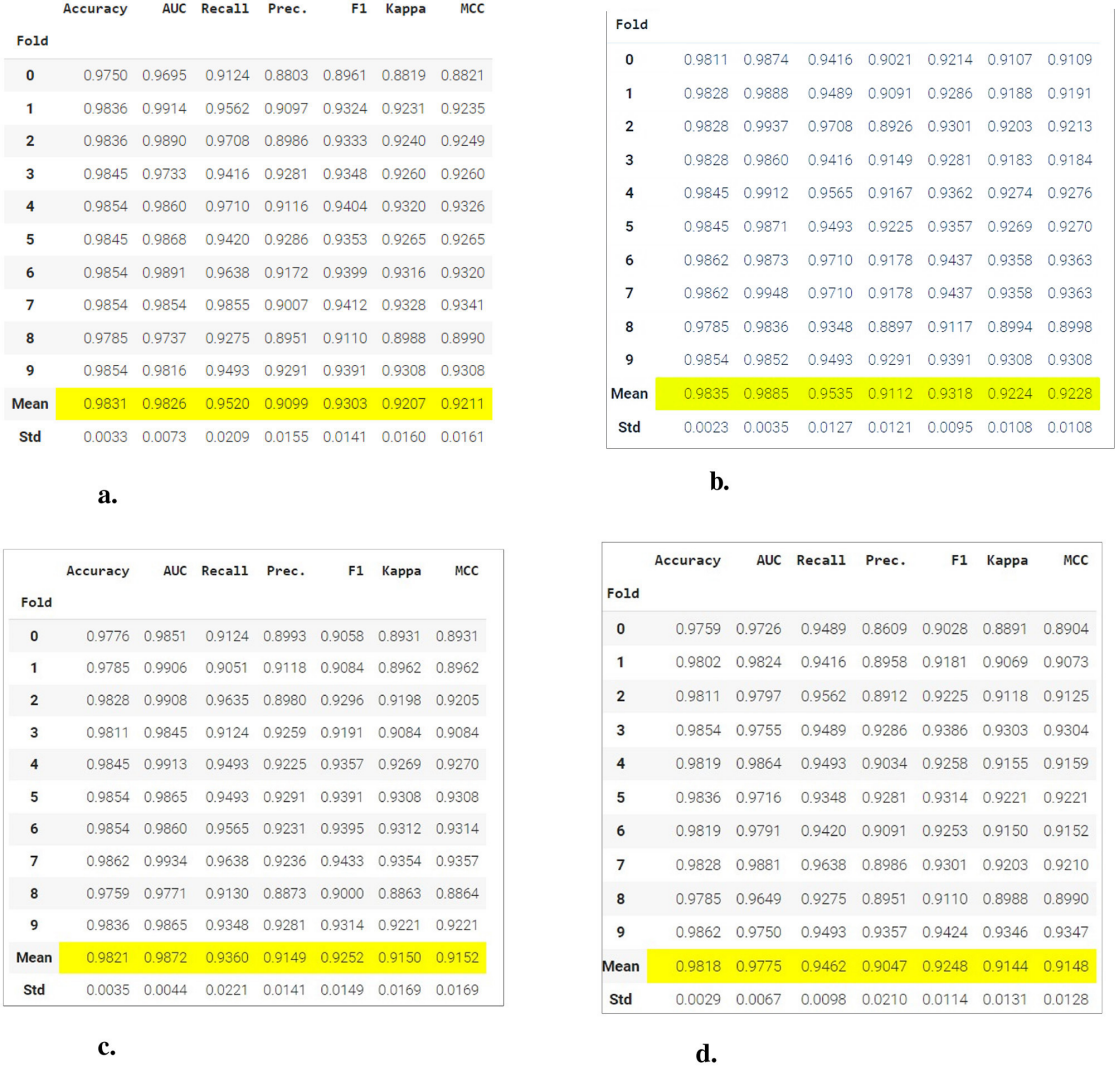
Comparison of performance metrics between different classification models using 10-fold cross-validation. **(A)** Gradient boosting classifier. **(B)** Light gradient boosting machine. **(C)** Extreme gradient boosting. **(D)** Logistic regression.

The Figure 7 presents a comparison of the results of various classification models in terms of multiple performance metrics, evaluated through 10-fold cross-validation. Within the figure, the tables highlight the average values of the key metrics in yellow, making it easy to compare the performance of the models in different classification scenarios.

### 5.11 Confusion matrix

**Figure 8:**
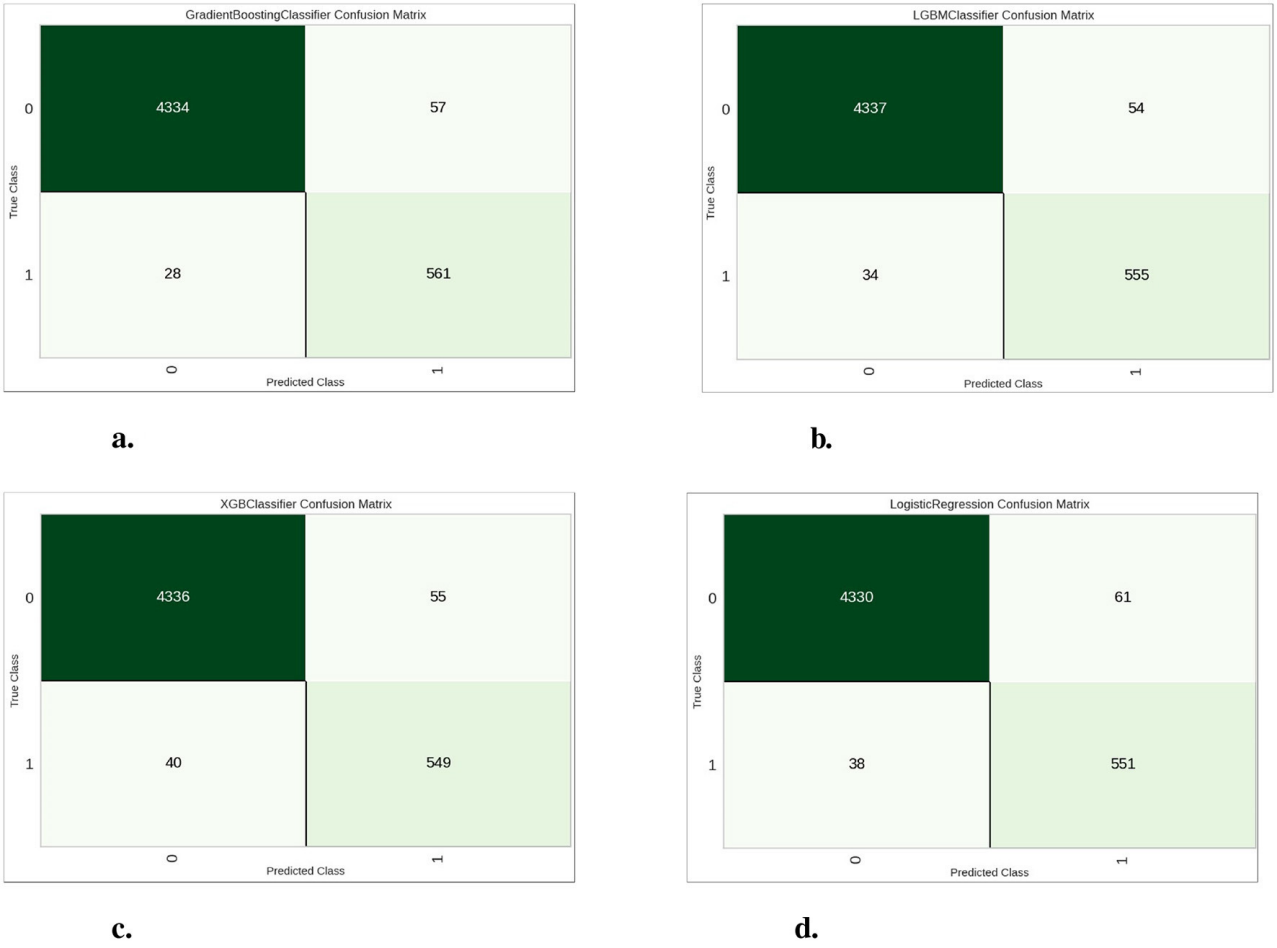
Confusion matrices for the four classification models. **(A)** Gradient boosting classifier. **(B)** Light gradient boosting machine. **(C)** Extreme gradient boosting. **(D)** Logistic regression.

Figure 8 shows the confusion matrices of four classifiers. The confusion matrices show how well each model does, and specifically the number of correct vs. incorrect predictions made by it in the validation data. Above, boosting models (i.e. Gradient Boosting, LightGBM and XGBoost) in general show better performance than the logistic regression having lower False positives errors as well false negatives. It clearly show that this dataset will works great with boosting models specially the Gradient Boosting model is better than others for classification accuracy score.

## 6 Constraints and challenges

### 6.1 Overfitting

One of the main challenges in building the model was the risk of overfitting, especially due to the use of a gradient boosting based algorithm, which is prone to overfitting the training data. To mitigate this problem, strategies such as cross-validation, hyperparameter optimization, and regularization by parameters such as maximum tree depth and learning rate were implemented.

### 6.2 Class imbalance

If not properly managed, typical metrics (e.g., accuracy) can be misleading, as a model that simply predicts the majority class will show high accuracy without actually capturing the minority class instances that really matter. To overcome this problem, it is recommended to use the Synthetic Minority Oversampling Technique (SMOTE), which consists of creating synthetic instances of the minority class using their nearest neighbors. This helps to avoid the even bigger problem of ensemble imbalance, as we do not want to lose information about the majority class and allow the model to learn better about both classes. In our case, after several tests evaluating the results using metrics such as the area under the precision-recall curve (AUC-ROC) and the F1 score, which are more appropriate in the presence of class imbalance than precision, we decided to keep the imbalanced data since the application of these techniques fell short of the metrics.

### 6.3 Real-time applicability

The model had a fairly good prediction, but could be expensive and slow in real production environments. Examples: XGBoost, LightGBM: Gradient boosting based models take a long time to train and can consume a lot of computational resources. One direction for future work would be to simplify the architecture to use a flatter model, or to explore the use of dimensionality reduction techniques to improve inference speed without sacrificing accuracy.

## 7 Results

This article was based on a case study of a B2B SME company for the creation of a B2B lead scoring model by analyzing data and applying classification supervised machine learning techniques, for its realization CRM data was used and the variables involved in lead conversion were explored.

This study coincides with the literature and the work of some authors who indicate that having a prediction about the probability that a potential customer has of buying, could positively impact B2B marketing (Priya, 2020), since the messages toward potential customers would be much more personalized and according to the stage of purchase in which they are (Nygård and Mezei, 2020) facilitating the conversion and being of help in the efficiency and digitization of the SME B2B company (Hofacker et al., 2020).

It is concluded that supervised machine learning based models can significantly improve the lead conversion rate and improve the effectiveness of marketing and sales activities in SME B2B companies.

The classification evaluation showed a good quality customer qualification model that is substantially superior to the one currently operated by the case company, and the model can be iteratively improved using feedback data. The study confirms the desirability of having a lead scoring model to guide the organization for efficient management of Sales and Marketing automation activities, aimed at achieving the business objectives set by the company.

### 7.1 Model performance

**Figure 9:**
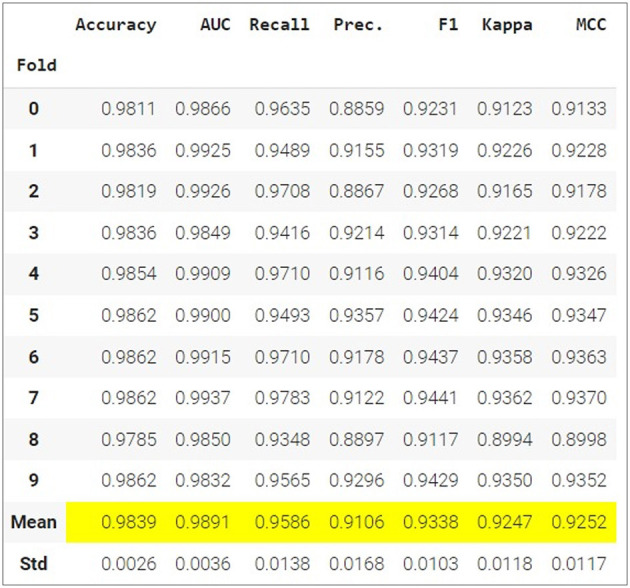
Gradient boosting classifier model tuning.

**Figure 10:**
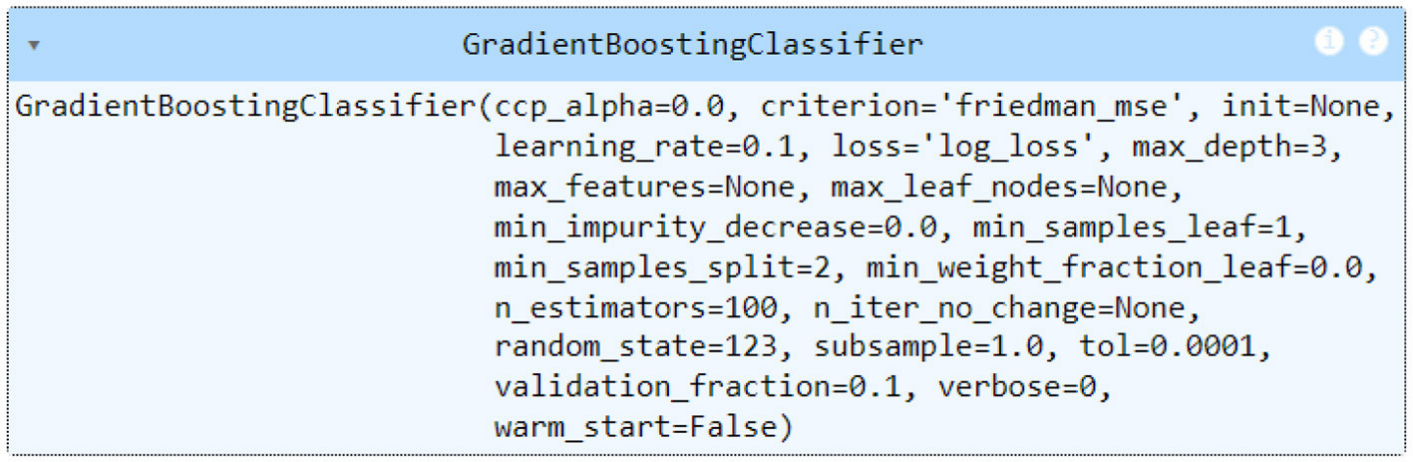
Setting the parameters of the Gradient boosting classifier model.

After comparing various classification models with PyCaret, the gradient boosting classifier emerged as the best performing model for this dataset. The model achieved an average accuracy of 98.39%, indicating a high level of predictive performance. The comprehensive evaluation metrics of PyCaret further corroborate the effectiveness of the model. These metrics suggest that the gradient boosting classifier not only accurately distinguishes between survivors and non-survivors but also maintains a good balance between precision and recall as evidenced by the F1 score. In addition, hyperparameter optimization is performed using Pycaret's tune function y the classification result can be seen in Figure 9. In Figure 10 we show the parameter configuration corresponding to the Gradient boosting classifier model.

**Figure 11:**
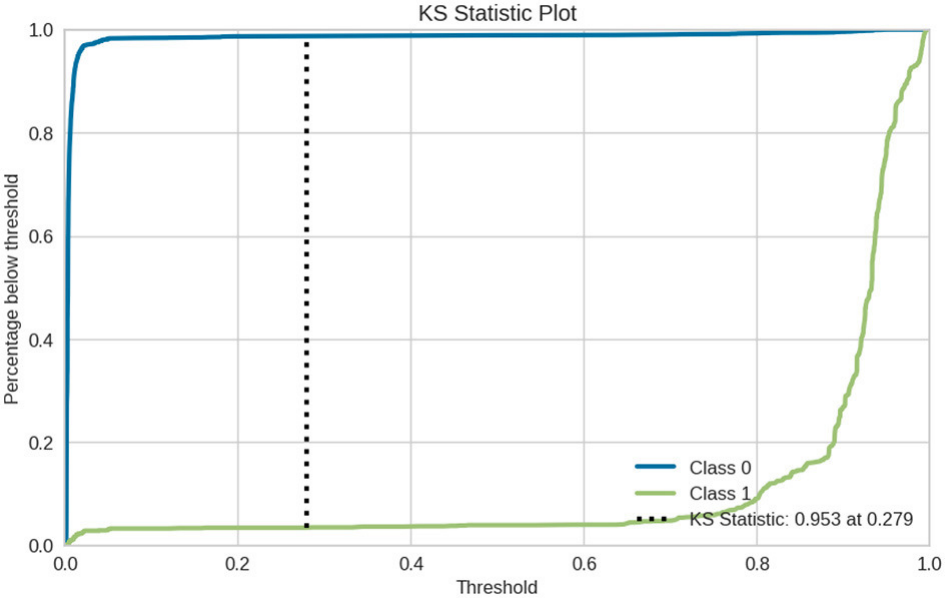
KS statistic plot for Gradient boosting classifier.

Figure 11 shows KS statistic (Kolmogorov–Smirnov), which is a measure of how well the binary classification model separates two classes by comparing it to the cumulative distribution of predicted probabilities for both Class 0 and Class 1. KS: 0.953: Therefore, the two classes are highly separated from each other A larger KS value indicates a model that is more effective at discriminating between the two classes. A value closer to 0.953 means the model is really good at separating Class 1 from Class 0 data points. Threshold (0.279): This is the threshold for which this separation of two class probabilities goes maximum. It is commonly used as the threshold value that optimizes it to create classification decisions.

Figure 10 shows the learning curve of Gradient boosting classifier. It compares the training and cross-validation scores based on how they evolve as the number of training instances increases. This curve has the following issues:

- No severe Underfitting issue in this model, as there is not a huge difference between training and validation scores.
- There is some little Overfitting in this model as well, it also happens at the start and shrinks as dataset becomes bigger.
- As we can see, the cross-validation score improves with more samples; hence having more data will probably improve performances of your model as well.
- A model that generalizes well will improve with more data on average.

**Figure 12:**
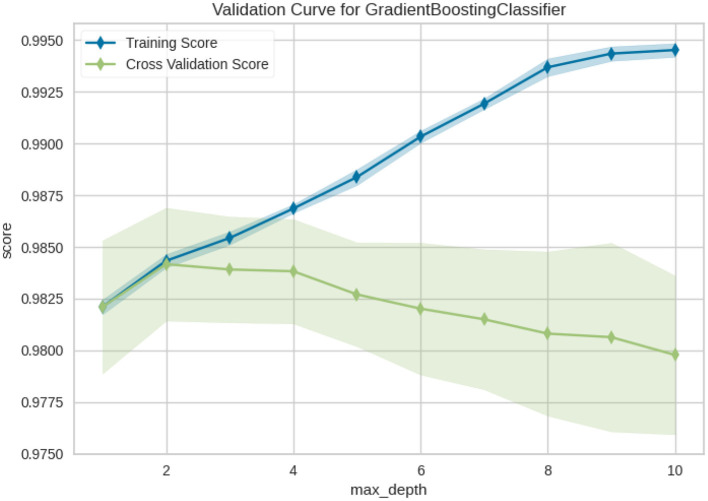
Validation curve of Gradient boosting classifier.

Figure 12 shows the validation curve for the Gradient boosting classifier model, from it is clear that max_depth influences performance not only on training data, but also in cross-validation. The blue line tells how much better our model can learn as trees get deep (Training Score) and shows almost a perfect score on the training set. This indicates that the model is capable of learning quite complicated patterns. The green curve (Cross Validation Score) is almost decreasing a bit but its at very high scores. This indicates that the model still performs well, even under large max_depth values.

### 7.2 Feature importance analysis

**Figure 13:**
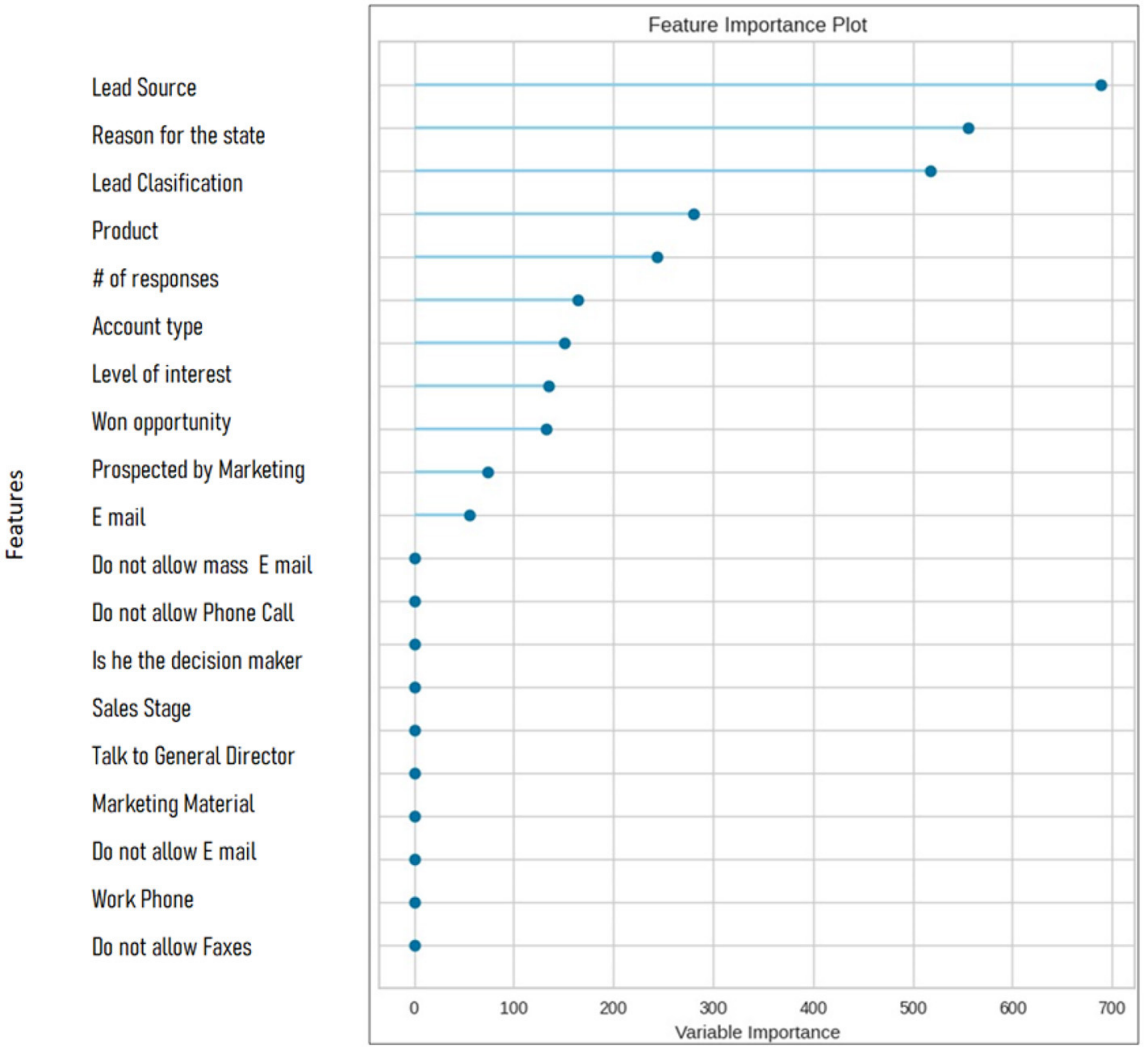
Feature importance.

Feature importance analysis was also performed in order to determine the most important features that contributed substantially on model predictions. The summary of the analysis is presented below: Variables in question are lead source, reason for state, categorization on leads and product (refer to Supplementary Appendix 5). PyCaret creates a feature importance chart to visually represent these observances which shows the significance of each contributing attribute in the model. Full list of feature importance is shown in Figure 13.

## 8 Discussion

This paper addresses a case study of a B2B company that was looking for a standardized and automated way to identify its priority leads in order to optimize the use of its sales reps' time spent on scoring B2B leads. Therefore, the purpose of this paper was to develop a systematic machine learning-based model to improve the accuracy of identifying high quality leads with the goal of prioritizing leads for sales reps in a B2B business case study.

The results showed that lead behavior and characteristics play an important role in building B2B lead scoring models. The top characteristics that contributed significantly to model predictions in this study were “Lead Source,” “Reason for State,” and “Lead Classification,” followed by “Product,” “Number of Responses,” “Account Type,” and “Interest Level. The “lead source” variable reveals the marketing strategy of the lead source, “number of responses” reflects the number of interactions with the company, “account type” reflects the segmentation of commercial, educational, and research customers, and “level of interest” shows the level of commitment of the potential customer to purchase a product or service from the company.

These characteristics reflect the behavior of potential customers when they interact with the company and answer the research question of this study, which are the most relevant characteristics of potential customers for the construction of the lead scoring model for this company. The above confirms the importance of consumer behavior theory in the development of lead scoring models and in the design of marketing strategies adapted to the interests and characteristics of customers (Solomon et al., 2012; Kotler et al., 2015; Solomon, 2020). Consumer behavior theory combined with market segmentation can improve the development of scoring systems (Goller et al., 2002).

This study is consistent with the literature and the work of some authors such as Priya (2020) and Espadinha-Cruz et al. (2021), who indicate that having a prediction of the probability of converting a potential customer could have a positive impact on the optimization of business processes and the overall marketing strategy, since it would allow sharing much more personalized messages aligned with the buying stage that potential customers are in Nygård and Mezei (2020), facilitating conversion and contributing to the efficiency and digitalization of the B2B SME company (Hofacker et al., 2020).

This study confirms previous research suggesting that lead scoring models are a central part of marketing strategy because they allow organizations to focus their efforts on the most likely and profitable opportunities (Lindahl, 2017). The findings of this study extend previous studies by Espadinha-Cruz et al. (2021), Stadlmann and Zehetner (2022), Gouveia and Costa (2022), and Jadli et al. (2023) on the importance of applying machine learning-based lead scoring systems.

The results of this study showed that the Gradient Boosting Classifier model has a superior performance compared to the other classifiers. The evaluation of the classification showed a good quality lead scoring model that is significantly superior to the one currently used by the company and that offers the possibility of being iteratively improved using feedback data compared to the existing traditional model. This supports the proposed alternative hypothesis that the use of machine learning-based lead scoring models will improve the accuracy of identifying high quality leads compared to traditional methods, thereby facilitating the prioritization of leads in B2B markets.

According to Nygård and Mezei (2020), Eitle and Buxmann (2019), and D'Haen and Van den Poel (2013), traditional lead scoring methods are ineffective and inaccurate, causing sales representatives to waste a lot of time on lead qualification activities. This research agrees with the authors (Armstrong, 2009) that it is not possible for sales representatives to serve an unlimited number of potential customers, which is why it is necessary for companies to segment their market to focus on the most appropriate ones and adapt their marketing strategies to build mutually satisfactory relationships (Sun, 2009).

The results of this study suggest that a machine learning-based lead scoring system can not only facilitate and optimize lead prioritization (Jadli et al., 2022) compared to traditional scoring methods, but also improve sales team efficiency by reducing the time required to assess lead quality, allowing sales teams to focus on opportunities with the highest potential, also impacting the marketing team by reducing the time involved in qualifying leads to outline efficient marketing strategies and content.

The results of this study expand the field of study of machine learning-based lead scoring systems and add value to the study by Wu et al. (2024b) by reinforcing their premise regarding the positive relationship that exists between scoring models and the reduction of lead conversion costs and the increase of high-quality leads, which could influence an optimization of the lead conversion rate and an increase in sales revenue.

This study agrees with Aggarwal (2014) and Jadli et al. (2022) on the value of artificial intelligence and answers the research questions on its influence on scoring models by demonstrating its functionality and influence on lead scoring through a case study of developing a lead scoring model, where supervised machine learning classification techniques were most appropriate to predict the probability of lead conversion in the B2B company of this case study (Paschen et al., 2019).

### 8.1 Academic and managerial implications

The results of this study have important practical implications for B2B marketing strategies. By prioritizing high-scoring leads, companies can allocate resources more effectively and increase sales team efficiency by focusing on the most viable and profitable opportunities. This approach not only improves conversion rates, but also optimizes the use of organizational resources. As there are few academic contributions in the literature on the success of lead scoring systems in B2B sales (Eitle and Buxmann, 2019; Nygård and Mezei, 2020; Espadinha-Cruz et al., 2021), this research will contribute to broaden the field of study of B2B lead scoring systems based on machine learning, adding a practical case of creating a lead scoring model to other previous studies by Espadinha-Cruz et al. (2021), Stadlmann and Zehetner (2022), Jadli et al. (2023), and Gouveia and Costa (2022), adding a practical case of developing a lead scoring model. In the business field, this research could be useful for managers seeking to improve the conversion and prioritization of potential customers, which could influence the reduction of B2B sales cycles and the increase of sales and therefore the revenue of organizations.

## 9 Conclusion

The use of machine learning-based lead scoring models improves the accuracy of identifying high-quality leads compared to traditional methods, making it easier to prioritize leads in B2B markets.

Below are the answers to the research questions and some key findings:

- Standardized and automated lead identification, replacing the traditional static model used by the case study company.
- Optimize the time B2B sales reps spend qualifying leads.
- According to the results of this research, the most relevant characteristics that significantly contributed to the predictions of the model in this study were “Lead Source,” “Reason for Condition,” and “Lead Classification,” followed by “Product,” “Number of Responses,” “Account Type,” and “Interest Level.”
- It concludes that AI-based models can significantly improve automated lead scoring, which would mean an improvement in lead conversion rate and the effectiveness of marketing and sales activities in B2B SMBs.
- The results of this research showed that the Gradient Boosting Classifier model was the best performing supervised machine learning classification algorithm in lead prediction compared to the other 15 classifiers applied.
- Integrating consumer behavior theory and market segmentation could further improve the effectiveness of lead scoring models.
- B2B lead conversion prediction was found to be a fertile area for research, as little attention has been paid to the success of B2B lead scoring models; therefore, the results of this study contribute to extending the theory and provide important practical implications for the design of machine learning-based lead scoring models with a focus on consumer behavior theory.

In conclusion, the results of this study underscore the importance of the lead scoring model as a strategic and essential tool in B2B marketing. Empirical evidence suggests that proper implementation of a machine learning-based lead scoring system not only facilitates and optimizes lead prioritization compared to traditional scoring methods, but also optimizes the time sales reps spend on lead scoring activities and enables marketers to develop customized marketing strategies aimed at achieving the company's business objectives. The study recommends that companies invest in developing and customizing machine learning-based lead scoring models that are aligned with their specific objectives to maximize their business effectiveness.

### 9.1 Limitations and possible future research

Although the model works correctly in scoring and prioritizing prospects, the recent development of this scoring model limits the evaluation of the results of the conversion rate of prospects to real customers qualified by this application, since the sales process of this B2B company has an average cycle of about 3 months. However, the embedded integration of this application into the company's customer relationship management system (Microsoft Dynamics) has just been completed, and the functioning of the implemented model is being evaluated in order to fine-tune the necessary adjustments. Consumer Behavior Theory was used to analyze the database in the CRM and was the basis for the selection and structure of the variables. A “buyer persona” model was also developed. However, the authors believe that its application could be further explored, as well as the market segmentation strategy. As the study is applied in the technology and services sector, it may show possible biases inherent to the industry in which the company involved in this research participates.

Lead scoring allows companies to obtain leads with a high probability of closing, which is a strategic issue in B2B companies, but the cycle may not be completely closed without evaluating sales reps so that companies can be sure they are delivering the highest quality lead to the most appropriate sales rep, possibly based on their skills, closing rate, and other aspects that could be explored in future research.

On the other hand, this study explores the importance of a B2B lead scoring model through a case study of an SME vendor in the technology sector. The authors believe it would be interesting to expand the evaluation of lead scoring models in other B2B verticals to identify similarities and trends.

## Data Availability

The datasets presented in this study can be found in online repositories. The names of the repository/repositories and accession number(s) can be found in the article/Supplementary material.
